# Associations of device‐measured sleep, sedentariness and physical activity with growth differentiation factor 15 in older adults

**DOI:** 10.1002/jcsm.12924

**Published:** 2022-02-07

**Authors:** Rosario Ortolá, Esther García‐Esquinas, Antonio Buño‐Soto, Verónica Cabanas‐Sánchez, David Martínez‐Gómez, Mercedes Sotos‐Prieto, Ellen A. Struijk, Francisco Félix Caballero, Esther Lopez‐Garcia, José R. Banegas, Fernando Rodríguez‐Artalejo

**Affiliations:** ^1^ Department of Preventive Medicine and Public Health Universidad Autónoma de Madrid Madrid Spain; ^2^ Cardiovascular and Nutritional Epidemiology Group IdiPAZ (La Paz University Hospital—Universidad Autónoma de Madrid) Madrid Spain; ^3^ CIBER of Epidemiology and Public Health (CIBERESP) Madrid Spain; ^4^ Department of Laboratory Medicine La Paz University Hospital‐IdiPaz Madrid Spain; ^5^ IMDEA Food Institute. CEI UAM + CSIC Madrid Spain; ^6^ Department of Environmental Health and Nutrition Harvard T.H. Chan School of Public Health Boston Massachusetts USA

**Keywords:** GDF‐15, Physical activity, Sedentary behaviours, Isotemporal substitution, Older adults

## Abstract

**Background:**

Growth differentiation factor 15 (GDF‐15) is a biomarker for chronic disease burden that might explain the health effects of sedentary behaviours (SBs) and physical activity (PA). We examined associations of device‐measured sleep, SB and PA, and time reallocations among them, with GDF‐15 in older adults.

**Methods:**

We used data from 2245 older adults participating in the Seniors‐ENRICA‐2 study. Wrist‐worn accelerometers were employed to ascertain total time in sleep, SB, light PA (LPA) and moderate‐to vigorous PA (MVPA). Associations between these activities and serum GDF‐15 levels were analysed using linear regression, including isotemporal substitution models for time reallocations among activities, and adjusted for potential confounders. Analyses were conducted separately in two groups (less active and more active individuals) according to the median total PA time.

**Results:**

In the less active participants, 30 min/day more of MVPA were related to lower levels of GDF‐15 when replacing sleep (fully adjusted mean percentage differences [95% confidence interval] in GDF‐15 of −9.2% [−13.2, −5.0]), SB (−9.8% [−13.6, −5.8]) and LPA (−5.8% [−11.1, −0.3]), whereas 30 min/day more of LPA were related to lower GDF‐15 when replacing both sleep (−3.6% [−6.1, −1.0]) and SB (−4.2% [−6.7, −1.7]). In the more active participants, 30 min/day more of MVPA were also associated with lower GDF‐15 when replacing sleep (−2.9% [−5.3, −0.3]), SB (−2.4% [−4.6, −0.2]) and LPA (−3.5% [−6.6, −0.3]), but no associations were found for more time in LPA. Spending more time in SB was associated with higher GDF‐15 levels only among those less active (1.9% [0.9, 2.9] per 30 min/day increment). Sleep time did not appear to be associated with GDF‐15.

**Conclusions:**

The MVPA was inversely associated with GDF‐15, with stronger associations at lower PA volumes. Also, more LPA and less SB time were linked to lower GDF‐15 in the less active individuals. This suggests that simply moving more and sitting less may reduce chronic disease burden in older adults.

## Introduction

Physical inactivity has a considerable detrimental impact on morbidity and premature mortality, and is responsible for a substantial economic burden.^1^, ^2^ This is of particular importance in older adults because, due to population aging, one third of the deaths and two thirds of the total burden of disease worldwide can be attributed to disorders in people aged 60 and above.^3^ In prospective studies conducted in this age group, physical activity (PA) has been associated with a substantially lower total mortality, irrespective of PA intensity,^4^ and has been identified as an important contributor to healthy aging.^5^ Conversely, sedentary behaviours (SBs) have been linked to increased risk of death, cardiometabolic conditions and poor mental health.^4^, ^6^ Besides, replacing SB with PA has been related to a lower risk of death^7^ and unhealthy aging,^8^ a better health‐related quality of life^9^ and improved cardiometabolic health,^10^, ^11^ with greater benefits for higher PA intensities. However, the biological mechanisms involved in the health effects of sleep, SB and PA are not completely understood, so there is a growing interest in identifying biomarkers that might reveal such mechanisms and explain these relationships.^12^

Growth differentiation factor 15 (GDF‐15) is a cytokine produced in response to inflammation, oxidative stress, hypoxia, telomere erosion, and oncogene activation.^13^, ^14^ It has been associated with all‐cause, cardiovascular and non‐cardiovascular death, independently of risk factors and other biomarkers related to mortality,^14^, ^15^ as well as with many chronic diseases, such as cardiovascular disease (CVD), type‐2 diabetes, neurodegenerative diseases, chronic renal disease and several cancers.^13^, ^14^, ^16^ Therefore, it has been recognized as a biomarker for unhealthy aging and chronic disease burden^13^ and could be a potential target for pharmacological or lifestyle interventions aimed to promote healthy aging. PA and SB are good candidates for these interventions, because of their modifiable nature and their strong influence on chronic disease.

Previous research on the association between PA and GDF‐15 has reported acute increases in plasma GDF‐15 levels after vigorous exercise bouts in young, healthy, active individuals,^17^, ^18^ but little is known about the effects of active or sedentary lifestyles in older adults. To our knowledge, only one study has analysed GDF‐15 concentrations in people of different ages and with different levels of PA, finding lower levels in active individuals vs. inactive ones at all ages. In older adults, GDF‐15 concentrations were approximately 45% and 50% lower in amateur endurance cyclists aged 61–71 years than in controls from the general population not actively exercising and patients with chronic lower limb mobility impairment of the same age, respectively; and about 25% lower in controls aged 72–83 years vs. patients of the same age.^19^ We aimed to delve into this relationship specifically in older adults by examining the associations of device‐measured sleep, SB and PA, and time reallocations among them, with GDF‐15. By using objective measures for both chronic disease burden and physical activities, and isotemporal substitution modelling, a statistical methodology that examines time reallocations among activities while controlling for the confounding effect of the remaining ones,^20^ we may be able to generate evidence that could influence public health recommendations regarding beneficial replacements among activities in older adults, a population group with a higher prevalence of chronic diseases and lower physical fitness.

## Methods

### Study design and participants

Data came from the baseline wave of the Seniors‐ENRICA‐2 cohort.^8^, ^21^ Participants were selected between 2015 and 2017 by stratified random sampling of all community‐dwelling individuals aged ≥65 years holding a national healthcare card and living in Madrid (Spain). A computer‐assisted telephone interview was performed to gather information on socio‐demographic data, lifestyle and morbidity, and two home visits were conducted to collect biological samples, perform a physical examination, attach a wrist accelerometer, and obtain a diet history. All participants provided written informed consent, and the Clinical Research Ethics Committee of ‘La Paz’ University Hospital in Madrid approved the study.

### Study variables

#### Sleep, sedentary behaviour and physical activity

Study participants wore an ActiGraph GT9X (ActiGraph Inc, Pensacola, FL, USA) accelerometer, attached to the non‐dominant wrist, for seven consecutive days and were asked to only remove it during bathing or swimming activities. Details on accelerometer data processing have been reported elsewhere.^21^ Time in SB and PA intensities was identified using previously proposed thresholds for the Euclidean Norm of the raw accelerations Minus One (ENMO): <45 m*g* for SB, 45–99 m*g* for light PA (LPA), and ≥100 m*g* for moderate‐to‐vigorous PA (MVPA),^22^ and sleep periods were detected with an automatized algorithm.^23^ Total PA time was calculated as the sum of time in LPA and MVPA. To investigate whether using only bouted SB or PA strengthened the associations, time in sedentary bouts ≥10 and ≥30 min, and in LPA and MVPA bouts ≥1 and ≥10 min was also ascertained, similarly to previous research in this field.^21^, ^24^, ^25^ PA bouts ≥1 min reflect bouted PA, that is, PA in bouts of any duration, but disregarding short duration movements. Bouts in each activity were considered when the 80% of the minimum required time met the threshold criteria. Only results from participants who wore the accelerometer ≥16 h/day during at least 4 days (at least three weekdays and at least one weekend day) were considered valid, because a wear time <16 h/day has been shown to augment underestimations of both SB and PA.^26^ Non‐wear time and time with abnormal high accelerations (i.e., ≥5.5 *g*) were imputed using the mean of the acceleration recorded for each participant during the corresponding time intervals.

#### GDF‐15

Fasting blood samples were collected from each participant in RST tubes with thrombin‐based clot activator and polymer gel (Becton Dickinson). Tubes were centrifuged at 3000 rpm for 10 min and serum was aliquoted, frozen at −80°C and stored in the Department of Preventive Medicine and Public Health at *Universidad Autónoma de Madrid*. Serum GDF‐15 was measured at the Department of Laboratory Medicine of ‘La Paz’ University Hospital by an electrochemiluminescence Elecsys® immunoassay method using a cobas® 6000 analyser (Roche Diagnostics). The inter‐assay coefficient of variation was 5.4% for a mean concentration of 7343 pg/mL and 7.7% for a mean concentration of 1428 pg/mL.

#### Potential confounders

We also collected information on sociodemographic and lifestyle characteristics including sex, age, educational level, tobacco smoking, and alcohol consumption. Food consumption and energy intake (kcal/day) were obtained from a validated diet history,^27^ and diet quality was estimated with the Mediterranean Diet Adherence Screener (MEDAS), ranging from 0 to 14, with higher scores indicating better adherence to the Mediterranean diet.^28^ The body mass index (BMI) was calculated as the weight (in kg) divided by the squared height (in m), both measured by standardized procedures.^29^ Blood pressure was measured three times under standardized conditions using validated devices, and the average of the 2nd and 3rd measurements was used for analyses. Fasting serum glucose, total cholesterol, HDL‐cholesterol and triglycerides were measured with colorimetric enzymatic methods using Atellica® solution (Siemens Healthineers), and LDL‐cholesterol was calculated with the Friedewald formula (LDL = total cholesterol − triglycerides/5 − HDL). Lastly, CVD was ascertained by asking the study participants if they had been previously diagnosed with acute myocardial infarction, stroke or heart failure, and diabetes mellitus was defined as current use of anti‐diabetic medication or a fasting blood glucose ≥126 mg/dL.

### Statistical analysis

The association of each activity with GDF‐15 was summarized with mean percentage differences (MPDs) in GDF‐15 per 30 min/day increment in sleep, SB, LPA or MVPA, and their 95% confidence interval (CI), obtained from linear regression models with log‐transformed GDF‐15 as the dependent variable; MPD in GDF‐15 were calculated by subtracting 1 from the exponentiated β coefficients in the regression models, and multiplying the result by 100. We built several models with incremental adjustment for potential confounders: Model 1 adjusted for sex, age and educational level; Model 2 further adjusted for tobacco smoking, alcohol consumption, MEDAS score and energy intake; and Model 3 further adjusted for BMI, glucose, LDL‐cholesterol, systolic blood pressure, CVD and diabetes. The association of time in PA and sedentary bouts with GDF‐15 was evaluated using the same statistical methods.

The association of time reallocation between sleep, SB, LPA or MVPA with GDF‐15 was summarized with MPD (95% CI) in GDF‐15 per 30 min/day replacement, obtained from isotemporal substitution linear regression models that included total time (24 h) and all activities simultaneously except the one being replaced. Regression models were adjusted as above.

Because the dose–response association between total PA time and GDF‐15 evaluated with restricted cubic splines was not linear (*P* < 0.001, supporting information *Figure* S1), and the direction of the association changed at approximately the study median of total PA time (3.44 h/day), analyses were stratified by categories of total PA time (≤ or > the median). Additional analyses stratifying by compliance with PA recommendations (< or ≥30 min/day of MVPA)^30^ were also performed. Also, to check the robustness of results, analyses were replicated excluding participants with CVD or diabetes, given that GDF‐15 is a strong biomarker of chronic disease burden. Lastly, we assessed whether the study associations differed for men and women by testing interaction terms defined as the product of time spent in each activity by categories of sex, but no statistically significant interactions were found. Statistical significance was set at two‐sided *P* value <0.05. Analyses were performed with Stata®, version 15 (StataCorp. 2017. Stata Statistical Software: Release 15. College Station, TX, USA: StataCorp LLC).

## Results

From the initial sample of 3273 individuals, we excluded 759 without valid accelerometry records, 207 without GDF‐15 measures and 62 with missing data on potential confounders of the study association. Thus, the analytical sample comprised 2245 individuals.

Study participants had a mean age of 71.6 years and 53.5% were women. Less active participants (those with a total PA time ≤ the median of 3.44 h/day) spent more time sleeping (33%) and in SB (56%), and less time in LPA (8%) and MVPA (2.5%) than the more active participants (31% of time sleeping, 49.5% in SB, 13% in LPA and 6% in MVPA). Times spent in the different activities according to the participants' socio‐demographic, lifestyle and clinical characteristics are presented in Table 1.

**Table 1:** Time spent in each activity by characteristics of study participants, stratified by total PA time

		Participants with low PA time^a^					Participants with high PA time^a^			
		Sleep	SB	LPA	MVPA		Sleep	SB	LPA	MVPA
	*n*	h/day	h/day	min/day	min/day	*n*	h/day	h/day	min/day	min/day
Total	1123	7.9 (1.2)	13.4 (1.3)	113.7 (31.2)	36.2 (19.4)	1122	7.5 (0.9)	11.8 (1.2)	186.8 (37.2)	85.5 (34.1)
Sex										
Men	571	7.8 (1.1)	13.6 (1.3)	107.3 (28.5)	41.2 (20.7)	473	7.3 (0.9)	12.0 (1.2)	173.4 (34.1)	95.1 (35.0)
Women	552	8.0 (1.1)*	13.3 (1.2)*	120.3 (32.4)*	31.0 (16.5)*	649	7.5 (0.9)*	11.7 (1.2)*	196.7 (36.3)*	78.5 (31.7)*
Age (years)										
65 to <70	413	7.9 (1.2)	13.3 (1.3)	114.6 (28.1)	43.0 (18.9)	586	7.5 (0.9)	11.8 (1.2)	184.4 (36.5)	92.2 (35.1)
≥70	710	7.9 (1.2)	13.5 (1.2)*	113.2 (32.2)	32.2 (18.6)*	536	7.4 (0.9)	11.9 (1.2)*	189.5 (37.8)*	78.1 (31.5)*
Educational level										
≤Primary	676	8.0 (1.3)	13.4 (1.3)	113.1 (32.5)	34.9 (19.7)	739	7.5 (0.9)	11.8 (1.2)	190.6 (37.9)	85.0 (33.8)
Secondary	219	7.8 (1.0)	13.5 (1.1)	115.3 (28.3)	39.1 (19.5)	198	7.5 (0.9)	11.8 (1.2)	184.3 (38.6)	87.1 (36.9)
University	228	7.7 (1.0)*	13.6 (1.2)*	113.9 (29.9)	37.1 (18.3)*	185	7.4 (0.9)	12.1 (1.1)*	174.7 (29.2)*	86.0 (32.4)
Tobacco smoking										
Non‐smoker	573	8.0 (1.2)	13.3 (1.3)	116.2 (31.6)	34.3 (18.7)	614	7.5 (0.9)	11.8 (1.2)	193.2 (38.0)	81.8 (32.7)
Former smoker	434	7.8 (1.1)	13.5 (1.2)	111.5 (30.1)	39.5 (20.4)	418	7.4 (0.9)	11.9 (1.2)	178.0 (34.2)	91.5 (36.1)
Current smoker	116	7.8 (1.4)*	13.6 (1.5)*	109.7 (32.2)*	32.8 (17.6)*	90	7.3 (1.0)	12.1 (1.3)*	184.2 (36.4)*	82.6 (31.1)*
Alcohol consumption										
Never drinker	231	8.1 (1.2)	13.3 (1.2)	113.4 (37.0)	29.7 (18.1)	200	7.5 (1.0)	11.8 (1.2)	194.5 (35.7)	79.1 (36.0)
Former drinker	74	8.1 (1.2)	13.3 (1.2)	112.7 (31.4)	31.8 (19.6)	64	7.5 (0.9)	11.8 (1.2)	189.0 (35.9)	85.1 (32.4)
Moderate drinker^b^	597	7.9 (1.2)	13.4 (1.3)	113.1 (29.7)	38.1 (18.9)	581	7.4 (0.9)	11.8 (1.2)	186.4 (37.7)	86.2 (33.6)
Heavy drinker	221	7.7 (1.1)*	13.6 (1.3)	112.7 (31.4)	31.8 (19.4)*	277	7.5 (0.9)	11.9 (1.2)	181.0 (35.9)*	88.8 (32.4)*
MEDAS score (0–14)										
≤6	397	7.9 (1.2)	13.5 (1.3)	111.8 (31.5)	35.2 (19.7)	357	7.5 (0.9)	11.8 (1.2)	189.2 (37.2)	84.0 (37.0)
7 to 8	513	7.9 (1.2)	13.4 (1.3)	114.4 (31.9)	36.2 (19.3)	485	7.5 (1.0)	11.9 (1.1)	185.4 (34.5)	85.1 (31.5)
≥9	213	7.9 (1.1)	13.4 (1.1)	115.6 (28.5)	37.8 (19.1)	280	7.4 (0.9)	11.8 (1.2)	186.3 (41.3)	88.2 (34.8)
Energy intake (kcal/day)										
<1800	383	8.0 (1.1)	13.4 (1.2)	114.6 (34.3)	31.9 (18.2)	363	7.5 (1.0)	11.8 (1.2)	192.1 (37.9)	78.7 (28.6)
1800 to 2050	372	8.0 (1.3)	13.4 (1.3)	113.1 (30.8)	36.4 (19.7)	382	7.5 (0.9)	11.9 (1.2)	185.5 (35.6)	85.9 (36.0)
>2050	368	7.8 (1.1)*	13.5 (1.2)	113.4 (28.0)	40.3 (19.6)*	377	7.4 (0.9)	11.9 (1.2)	183.1 (37.5)*	91.6 (36.0)*
BMI (kg/m^2^)										
<25	259	8.1 (1.2)	13.2 (1.3)	116.1 (30.6)	37.0 (18.9)	353	7.6 (0.9)	11.6 (1.2)	191.9 (37.1)	86.1 (35.0)
25 to <30	517	7.9 (1.1)	13.4 (1.2)	114.2 (30.4)	39.6 (20.4)	558	7.4 (0.9)	11.9 (1.2)	184.0 (39.0)	87.4 (34.1)
≥30	347	7.8 (1.2)	13.7 (1.3)*	111.2 (32.6)	30.3 (17.0)*	211	7.3 (1.0)*	12.1 (1.2)*	186.0 (34.4)*	79.4 (32.1)*
Cardiovascular disease^c^										
No	1073	7.9 (1.2)	13.4 (1.2)	114.1 (30.5)	36.5 (19.5)	1093	7.5 (0.9)	11.8 (1.2)	186.9 (37.3)	85.7 (34.1)
Yes	50	8.1 (1.7)	13.5 (1.7)	104.5 (42.5)*	29.4 (17.8)*	29	7.5 (1.1)	12.0 (1.3)	185.8 (31.1)	78.6 (34.3)
Diabetes										
No	852	7.9 (1.2)	13.3 (1.2)	116.3 (29.2)	37.7 (19.3)	957	7.5 (0.9)	11.8 (1.2)	188.1 (36.8)	85.7 (34.4)
Yes	271	7.8 (1.3)	13.7 (1.3)*	105.7 (35.5)*	31.2 (19.1)*	165	7.4 (0.9)	12.0 (1.2)	179.8 (38.7)*	84.4 (32.8)
SBP (mmHg)										
<130	439	7.9 (1.2)	12.9 (1.5)	115.1 (32.2)	35.7 (19.3)	491	7.4 (0.9)	11.9 (1.2)	188.1 (36.9)	85.8 (34.2)
≥130	684	7.9 (1.2)	12.4 (1.4)	112.8 (30.5)	36.5 (19.6)	631	7.5 (0.9)	11.8 (1.2)	185.9 (37.4)	85.3 (34.1)
Serum glucose (mg/dL)										
<100	666	8.0 (1.2)	13.3 (1.2)	117.0 (29.0)	38.1 (19.2)	776	7.5 (0.9)	11.8 (1.2)	189.4 (38.2)	85.1 (34.0)
≥100	457	7.9 (1.2)	13.6 (1.3)*	108.9 (33.6)*	33.4 (19.4)*	346	7.4 (0.9)	11.9 (1.2)*	181.1 (34.1)*	86.4 (34.4)
Serum LDL‐cholesterol (mg/dL)										
<100	413	7.8 (1.3)	13.6 (1.3)	109.2 (33.2)	33.6 (19.5)	291	7.4 (0.9)	12.0 (1.2)	183.9 (38.1)	81.7 (33.8)
≥100	710	8.0 (1.1)	13.3 (1.2)*	116.3 (29.6)*	37.6 (19.2)*	831	7.5 (0.9)	11.8 (1.2)*	187.9 (36.8)	86.8 (34.2)*

BMI, body mass index; LPA, light physical activity; MEDAS, Mediterranean Diet Adherence Screener; MVPA, moderate‐to‐vigorous physical activity; PA. physical activity; SB, sedentary behaviour; SBP, systolic blood pressure. Values are means (standard deviations).

* *P* < 0.05.

^a^ Low PA time: total PA time ≤3.44 h/day; high PA time: total PA time >3.44 h/day.

^b^ Moderate drinker: <10 g/day in women and <20 g/day in men.

^c^ Including acute myocardial infarction, stroke and congestive heart failure.

In analyses examining associations between each activity and GDF‐15 without taking into account the other activities, spending more time in SB was associated with higher GDF‐15 levels only among those in the less physically active group, with fully adjusted MPD (95% CI) per 30 min/day increment of 1.9% (0.9, 2.9) (Table 2). This association did not vary when only time accumulated in sedentary bouts ≥10 or ≥30 min was counted (Table 3). On the contrary, spending 30 min/day more in LPA was associated with lower GDF‐15 levels [−5.9% (−8.1, −3.6)], as well as more time in LPA bouts ≥1 min [−5.9 (−11.2, −0.2)], again only in those less active. Spending 30 min/day more in MVPA was also associated with lower GDF‐15 both in less active participants [−11.8% (−15.3, −8.1)] and more active ones, although to a much lesser extent [−2.4% (−4.6, −0.3)] (Table 2). Lower GDF‐15 levels were also found for more time spent in MVPA bouts ≥1 min both in less active individuals [−10.4% (−14.8, −5.9)] and more active ones [−4.0% (−6.6, −1.4)], as well as for more time spent in MVPA bouts ≥10 min [−9.3% (−16.6, −1.3) in the less active group, and −4.0% (−7.6, −0.4) in the more active group] (Table 3). Sleep time did not appear to be associated with GDF‐15 levels.

**Table 2:** Association of time spent in each activity with GDF‐15, stratified by total PA time

	Participants with low PA time^a^		Participants with high PA time^a^		p for interaction
	*n* = 1123		*n* = 1122		
Sleep					
Model 1	−0.4 (−1.5, 0.7)		0.3 (−1.2, 1.8)		0.46
Model 2	−0.5 (−1.6, 0.7)		0.4 (−1.0, 1.9)		0.33
Model 3	−0.1 (−1.1, 0.9)		0.6 (−0.8, 1.9)		0.44
SB					
Model 1	3.1 (2.0, 4.3)***		0.5 (−0.6, 1.7)		0.001
Model 2	3.0 (1.9, 4.1)***		0.4 (−0.8, 1.5)		0.001
Model 3	1.9 (0.9, 2.9)***		0.0 (−1.0, 1.1)		0.01
LPA					
Model 1	−8.8 (−11.2, −6.4)***		−0.3 (−2.5, 1.9)		<0.001
Model 2	−8.4 (−10.7, −6.0)***		−0.3 (−2.5, 1.9)		<0.001
Model 3	−5.9 (−8.1, −3.6)***		0.4 (−1.6, 2.5)		<0.001
MVPA					
Model 1	−17.6 (−21.1, −14.0)***		−3.6 (−5.9, −1.3)**		<0.001
Model 2	−16.5 (−20.0, −12.8)***		−3.3 (−5.6, −0.9)**		<0.001
Model 3	−11.8 (−15.3, −8.1)***		−2.4 (−4.6, −0.3)*		<0.001

GDF‐15, growth differentiation factor 15; LPA, light physical activity; MVPA, moderate‐to‐vigorous physical activity; PA, physical activity; SB, sedentary behaviour.

Values are mean percentage differences ([exponentiated differences in log‐transformed values of GDF‐15 − 1] × 100) per 30 min/day increment (95% confidence interval).

Model 1: Linear regression model adjusted for sex, age, and educational level (primary or less, secondary, or university).

Model 2: As Model 1 and further adjusted for smoking status (never, former or current), alcohol consumption (never, moderate, heavy or former), energy intake (kcal/day) and Mediterranean Diet Adherence Screener (MEDAS) score.

Model 3: As Model 2 and further adjusted for body mass index (kg/m^2^), serum glucose (mg/dL), serum LDL‐cholesterol (mg/dL), systolic blood pressure (mmHg), cardiovascular disease (including acute myocardial infarction, stroke and congestive heart failure) and diabetes.

* *P* < 0.05.

** *P* < 0.01.

*** *P* < 0.001.

^a^ Low PA time: total PA time ≤3.44 h/day; high PA time: total PA time >3.44 h/day.

**Table 3:** Association of time accumulated in bouts of each activity with GDF‐15, stratified by total PA time

	Participants with low PA time^a^	Participants with high PA time^a^	*P* for interaction
	*n* = 1123	*n* = 1122	
Time in sedentary bouts ≥10 min	1.8 (1.1, 2.6)***	−0.2 (−0.9, 0.6)	<0.001
Time in sedentary bouts ≥30 min	1.9 (1.2, 2.5) ***	−0.2 (−0.9, 0.5)	<0.001
Time in LPA bouts ≥1 min	−5.9 (−11.2, −0.2)*	−1.2 (−4.7, 2.5)	0.16
Time in LPA bouts ≥10 min	−8.8 (−21.6, 6.0)	−8.3 (−16.9, 1.1)	0.95
Time in MVPA bouts ≥1 min	−10.4 (−14.8, −5.9)***	−4.0 (−6.6, −1.4)**	0.01
Time in MVPA bouts ≥10 min	−9.3 (−16.6, −1.3)*	−4.0 (−7.6, −0.4)*	0.22

GDF‐15, growth differentiation factor 15; LPA, light physical activity; MVPA, moderate‐to‐vigorous physical activity; PA, physical activity.

Values are mean percentage differences ([exponentiated differences in log‐transformed values of GDF‐15 − 1] × 100) per 30 min/day increment (95% confidence interval).

* *P* < 0.05.

** *P* < 0.01.

*** *P* < 0.001.

^a^ Low PA time: total PA time ≤3.44 h/day; high PA time: total PA time >3.44 h/day.

Linear regression model adjusted for sex, age, educational level (primary or less, secondary, or university), smoking status (never, former, or current), alcohol consumption (never, moderate, heavy, or former), energy intake (kcal/day), Mediterranean Diet Adherence Screener (MEDAS) score, body mass index (kg/m^2^), serum glucose (mg/dL), serum LDL‐cholesterol (mg/dL), systolic blood pressure (mmHg), cardiovascular disease (including acute myocardial infarction, stroke and congestive heart failure) and diabetes.

In the less active participants, 30 min/day more of MVPA were related to lower levels of GDF‐15 when replacing sleep [−9.2% (−13.2, −5.0)], SB [−9.8% (−13.6, −5.8)] and LPA [−5.8% (−11.1, −0.3)], whereas 30 min/day more of LPA were related to lower GDF‐15 when replacing both sleep [−3.6% (−6.1, −1.0)] and SB [−4.2% (−6.7, −1.7)]. In the more active participants, 30 min/day more of MVPA were also associated with lower GDF‐15 when replacing sleep [−2.9% (−5.3, −0.3)], SB [−2.4% (−4.6, −0.2)] and LPA [−3.5% (−6.6, −0.3)], although these associations were much smaller than those observed in the less active group. However, no associations were found for more time in LPA (Table 4).

**Table 4:** Association of isotemporal replacement of activities with GDF‐15, stratified by total PA time

	Participants with low PA time^a^	Participants with high PA time^a^	*P* for
	*n* = 1123	*n* = 1122	interaction
Sleep → SB			
Model 1	1.2 (0.0, 2.4)*	−0.2 (−1.7, 1.3)	0.14
Model 2	1.2 (0.1, 2.4)*	−0.3 (−1.8, 1.1)	0.10
Model 3	0.7 (−0.4, 1.7)	−0.4 (−1.8, 0.9)	0.20
Sleep → LPA			
Model 1	−5.1 (−7.7, −2.4)***	0.4 (−2.0, 3.0)	0.003
Model 2	−4.9 (−7.5, −2.2)**	0.3 (−2.1, 2.8)	0.005
Model 3	−3.6 (−6.1, −1.0)**	0.7 (−1.6, 3.0)	0.01
Sleep → MVPA			
Model 1	−13.7 (−17.7, −9.4)***	−3.7 (−6.3, −0.9)**	<0.001
Model 2	−12.6 (−16.7, −8.3)***	−3.5 (−6.1, −0.7)*	<0.001
Model 3	−9.2 (−13.2, −5.0)***	−2.9 (−5.3, −0.3)*	0.007
SB → LPA			
Model 1	−6.2 (−8.8, −3.5)***	0.6 (−1.6, 3.0)	<0.001
Model 2	−6.0 (−8.6, −3.3)***	0.7 (−1.6, 2.9)	<0.001
Model 3	−4.2 (−6.7, −1.7)**	1.1 (−1.0, 3.2)	0.001
SB → MVPA			
Model 1	−14.7 (−18.6, −10.7)***	−3.5 (−5.8, −1.1)**	<0.001
Model 2	−13.6 (−17.6, −9.5)***	−3.1 (−5.4, −0.8)*	<0.001
Model 3	−9.8 (−13.6, −5.8)***	−2.4 (−4.6, −0.2)*	0.001
LPA → MVPA			
Model 1	−9.1 (−14.6, −3.2)**	−4.1 (−7.4. –0.6)*	0.003
Model 2	−8.1 (−13.7, −2.2)**	−3.8 (−7.1. –0.3)*	0.18
Model 3	−5.8 (−11.1, −0.3)*	−3.5 (−6.6. –0.3)*	0.45

GDF‐15, growth differentiation factor 15; LPA, light physical activity; MVPA, moderate‐to‐vigorous physical activity; PA, physical activity; SB, sedentary behaviour.

Values are mean percentage differences ([exponentiated differences in log‐transformed values of GDF‐15 − 1] × 100) per 30 min/day replacement (95% confidence interval).

Model 1: Linear regression model including total time (24 h) and all activities (sleep, SB, LPA, and MVPA) except the one being replaced, and adjusted for sex, age, and educational level (primary or less, secondary, or university).

Model 2: As Model 1 and further adjusted for smoking status (never, former or current), alcohol consumption (never, moderate, heavy or former), energy intake (kcal/day) and Mediterranean Diet Adherence Screener (MEDAS) score.

Model 3: As Model 2 and further adjusted for body mass index (kg/m^2^), serum glucose (mg/dL), serum LDL‐cholesterol (mg/dL), systolic blood pressure (mmHg), cardiovascular disease (including acute myocardial infarction, stroke and congestive heart failure) and diabetes.

* *P* < 0.05.

** *P* < 0.01.

*** *P* < 0.001.

^a^ Low PA time: total PA time ≤3.44 h/day; high PA time: total PA time >3.44 h/day.

Analyses excluding participants with CVD or diabetes rendered similar results, although most associations attenuated slightly, and the association with lower GDF‐15 levels of increasing time in MVPA at the expense of LPA did not remain in any of the groups, neither did the association when replacing sleep with MVPA in the more active group (*Tables* S1–S3). Lastly, consistent results were obtained when stratifying by compliance with PA recommendations. Specifically, in participants not meeting PA recommendations, much larger associations with lower GDF‐15 levels (between −25% and −40%) were found for increasing time in MVPA or in MVPA bouts ≥1 min, and for replacing sleep, SB or LPA with MVPA. In those meeting PA recommendations, no association was apparent for replacing LPA with MVPA (*Tables* S4–S6).

## Discussion

In this sample of older adults in Spain, MVPA was associated with lower GDF‐15 irrespective of the participants' PA time. This association was evident for more time in MVPA and in MVPA bouts ≥1 or ≥10 min, and for substituting MVPA for sleep, SB or LPA, and was stronger in the less than in the more physically active participants. Besides, only in the less active group, more time in LPA, and substituting LPA for sleep or SB were also related to lower GDF‐15, whereas more time spent in SB (total time or time in sedentary bouts ≥10 or ≥30 min) was associated with higher GDF‐15 levels. However, in the more active participants, LPA or SB variables were not related to GDF‐15. The found associations have clinical relevance because in our study, each 24% increment in GDF‐15 was associated with a 0.74‐point higher health deficit accumulation index, which corresponds to the 1 year increase in this measure of unhealthy aging observed in a very similar cohort (Seniors‐ENRICA‐1).^31^ Thus, in the less active group, replacing 30 min/day of SB with MVPA or LPA would delay unhealthy aging by about 5 or 2 months, respectively, and replacing 30 min/day of LPA with MVPA would delay unhealthy aging by about 3 months.

The health benefits of MVPA are clear,^1^, ^4^, ^32^ and most guidelines recommend that adults, including older adults, practice at least 150 min/week of MVPA.^33^, ^34^, ^35^ The most recent recommendation from the World Health Organization is to do at least 150–300 min/week of moderate aerobic PA or at least 75–150 min of vigorous aerobic PA, or an equivalent combination of both, but these times may be increased to gain additional health benefits.^36^ However, even low volumes of MVPA have been linked to health benefits, including lower death risk, with the greatest relative benefits being observed when increasing PA from no or minimal activity to small amounts, well below the recommended ones.^32^, ^37^ Also debated is the suggestion to accumulate this activity in bouts of at least 10 min, because there is some evidence that both short and long bouts of MVPA have beneficial effects on cardiometabolic health, multimorbidity, successful aging and mortality,^32^, ^38^, ^39^, ^40^ so the most recent PA guidelines do not require that PA should be performed in bouts of sufficient duration.^33^, ^34^, ^35^ The lower chronic disease burden estimated with GDF‐15 found in our study for higher levels of MVPA is in line with the existing evidence. Importantly, our findings also suggest that the less physically active an individual is, the more would benefit from increasing MVPA, and that total time or time accumulated in short bouts could be as favourable as time accrued in long bouts. Therefore, although many older adults are not able to meet the recommended goal, they should be encouraged to include some MVPA in their daily lives.

Growing evidence also supports the health benefits of LPA, including a lower risk of obesity, CVD, cardiometabolic risk factors, unhealthy aging and mortality.^8^, ^41^, ^42^ SB time, however, has been related to several poor health outcomes,^6^ and replacing SB with PA of any intensity has been favourably associated with mortality, health‐related quality of life, adiposity and cardiometabolic risk factors and conditions in middle‐aged and older adults.^4^, ^5^, ^6^, ^7^, ^8^, ^9^, ^10^, ^11^ Even replacing sitting with standing has shown health benefits.^7^ Therefore, LPA can be a means to break up prolonged periods of SB, especially in those less active and/or with low exertional capacities, in line with our findings in less active participants. In this sense, in less active individuals, devoting more time to LPA would reflect a less sedentary and more active lifestyle, with the resultant health benefits. However, because high levels of MVPA appear to reduce or even remove some of the detrimental health effects of SB,^43^ we did not find any associations between sedentariness variables and GDF‐15 in the more active group, suggesting that SB is not harmful when accompanied by high levels of MVPA, especially in older adults.

The main limitation of this study is its cross‐sectional design, which does not allow us to draw causal inferences. However, because the associations remained after excluding individuals with CVD or diabetes, it is unlikely that the disease burden captured by GDF‐15 would have substantially influenced time spent in the different activities. Also, we are assessing theoretical changes in time allocated to these activities, and not actual ones; and GDF‐15 may have been subjected to some measurement error, which might have attenuated the true associations. Moreover, as in any observational study, we cannot entirely rule out residual confounding, despite adjusting for many potential confounders. Lastly, this study was conducted in older adults of a Mediterranean country, with a characteristic and distinct lifestyle, so our results may not be generalizable to other populations.

In conclusion, more MVPA was associated with lower levels of GDF‐15 in older adults, with stronger associations at lower PA volumes, whereas more LPA and less SB time were associated with lower GDF‐15 only in less physically active participants. These findings suggest that increasing time in MVPA may reduce the chronic disease burden captured by GDF‐15 and thus promote healthy aging, counteracting even the increased disease burden associated with sedentariness. In addition, in those individuals with lower PA capabilities for whom increasing MVPA may be unrealistic, some benefit could be obtained by reducing sedentary time and replacing it with activities of lower intensity, such as walking or household chores, which is a more attainable strategy. However, our results should be confirmed by prospective studies examining actual changes in the different activities.

## Conflict of interest

The authors declare that they have no conflicts of interest.

## Supporting information


**Figure S1.** Dose–response association of total PA time with GDF‐15
**Table S1.** Association of time spent in each activity with GDF‐15 excluding participants with cardiovascular disease^a^ or diabetes, stratified by total PA time
**Table S2.** Association of time accumulated in bouts of each activity with GDF‐15 excluding participants with cardiovascular disease^a^ or diabetes, stratified by total PA time
**Table S3.** Association of isotemporal replacement of activities with GDF‐15 excluding participants with cardiovascular disease^a^ or diabetes, stratified by total PA time
**Table S4.** Association of time spent in each activity with GDF‐15, stratified by compliance with PA recommendations (≥30 min/day of MVPA)
**Table S5.** Association of time accumulated in bouts of each activity with GDF‐15, stratified by compliance with PA recommendations (≥30 min/day of MVPA)
**Table S6.** Association of isotemporal replacement of activities with GDF‐15, stratified by compliance with PA recommendations (≥30 min/day of MVPA)
