# Supplementary material for: Associations of device‐measured sleep, sedentariness and physical activity with growth differentiation factor 15 in older adults
Source: J Cachexia Sarcopenia Muscle. 2022 Feb 7;13(2):1003–12. doi: 10.1002/jcsm.12924 (PMC8977966; doi:10.1002/jcsm.12924)
Supplement: Supplementary file 1 — Figure S1. Dose–response association of total PA time with GDF‐15 Table S1. Association of time spent in each activity with GDF‐15 excluding participants with cardiovascular diseasea or diabetes, stratified by total PA time Table S2. Association of time accumulated in bouts of each activity with GDF‐15 excluding participants with cardiovascular diseasea or diabetes, stratified by total PA time Table S3. Association of isotemporal replacement of activities with GDF‐15 excluding participants with cardiovascular diseasea or diabetes, stratified by total PA time Table S4. Association of time spent in each activity with GDF‐15, stratified by compliance with PA recommendations (≥30 min/day of MVPA) Table S5. Association of time accumulated in bouts of each activity with GDF‐15, stratified by compliance with PA recommendations (≥30 min/day of MVPA) Table S6. Association of isotemporal replacement of activities with GDF‐15, stratified by compliance with PA recommendations (≥30 min/day of MVPA) [file JCSM-13-1003-s001.pdf]

Figure S1. Dose-response association of total PA time with GDF-15

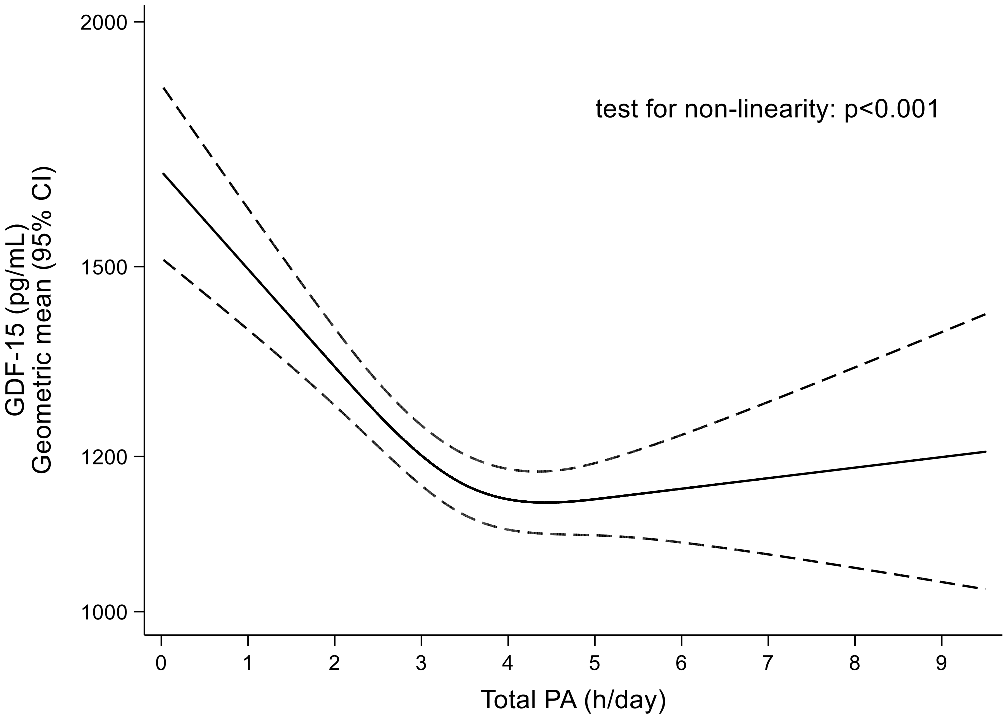

GDF-15, growth differentiation factor 15; PA, physical activity.

Restricted cubic spline (knots at the 10th, 50th and 90th percentile) from a linear regression model adjusted for sex, age, educational level (primary or less, secondary, or university), smoking status (never, former, or current), alcohol consumption (never, moderate, heavy, or former), energy intake (kcal/day), Mediterranean Diet Adherence Screener (MEDAS) score, body mass index (kg/m^2), serum glucose (mg/dL), serum LDL-cholesterol (mg/dL), systolic blood pressure (mmHg), cardiovascular disease (including acute myocardial infarction, stroke and congestive heart failure) and diabetes.

Table S1. Association of time spent in each activity with GDF-15 excluding participants with cardiovascular disease[a] or diabetes, stratified by total PA time

	**Participants with low PA time[b]** n=822	**Participants with high PA time[b]** n=932	**P for interaction**
**Sleep**			
Model 1	–0.1 (–1.2, 1.1)	0.2 (–1.5, 1.2)	0.92
Model 2	–0.1 (–1.2, 1.1)	0.0 (–1.3, 1.4)	0.90
Model 3	0.0 (–1.1, 1.2)	0.1 (–1.2, 1.5)	0.88
**SB**			
Model 1	1.7 (0.6, 2.8)**	0.7 (–0.3, 1.8)	0.20
Model 2	1.5 (0.4, 2.6)**	0.5 (–0.5, 1.6)	0.22
Model 3	1.4 (0.3, 2.5)*	0.5 (–0.5, 1.6)	0.27
**LPA**			
Model 1	–6.2 (–8.7, –3.5)***	–0.3 (–2.3, 1.8)	**<0.001**
Model 2	–5.5 (–8.0, –2.9)***	–0.4 (–2.4, 1.7)	**0.002**
Model 3	–5.4 (–7.9, –2.7)***	–0.4 (–2.4, 1.6)	**0.003**
**MVPA**			
Model 1	–10.4 (–14.1, –6.4)***	–3.1 (–5.2, –0.9)**	**0.001**
Model 2	–8.7 (–12.5, –4.7)***	–2.6 (–4.7, –0.5)*	**0.006**
Model 3	–8.5 (–12.4, –4.5)***	–2.6 (–4.7, –0.5)*	**0.009**

GDF-15, growth differentiation factor 15; LPA, light physical activity; MVPA, moderate-to-vigorous physical activity; PA, physical activity; SB, sedentary behavior.

Values are mean percentage differences ([exponentiated differences in log-transformed values of GDF-15 – 1] x 100) per 30 min/day increment (95% confidence interval).

Model 1: Linear regression model adjusted for sex, age, and educational level (primary or less, secondary, or university).

Model 2: As model 1 and further adjusted for smoking status (never, former, or current), alcohol consumption (never, moderate, heavy, or former), energy intake (kcal/day) and Mediterranean Diet Adherence Screener (MEDAS) score.

Model 3: As model 2 and further adjusted for body mass index (kg/m^2), serum glucose (mg/dL), serum LDL-cholesterol (mg/dL), and systolic blood pressure (mmHg).

* $P < 0.05$.

** $P < 0.01$

*** $P < 0.001$.

[a] Including acute myocardial infarction, stroke and congestive heart failure.

[b] Low PA: total PA time ≤3.44 h/day; high PA: total PA time >3.44 h/day.

Table S2. Association of time accumulated in bouts of each activity with GDF-15 excluding participants with cardiovascular disease[a] or diabetes, stratified by total PA time

	**Participants with low PA time**[b] n=822	**Participants with high PA time**[b] n=932	**P for interaction**
**Time in sedentary bouts ≥10 min**	1.4 (0.6, 2.2)**	0.1 (–0.6, 0.9)	**0.02**
**Time in sedentary bouts ≥30 min**	1.5 (0.7, 2.2)***	–0.0 (–0.7, 0.7)	**0.003**
**Time in LPA bouts ≥1 min**	–5.8 (–11.6, 0.3)	–2.2 (–5.7, 1.4)	0.31
**Time in LPA bouts ≥10 min**	–4.8 (–19.2, 12.3)	–7.6 (–15.9, 1.4)	0.75
**Time in MVPA bouts ≥1 min**	–7.5 (–12.0, –2.7)**	–4.2 (–6.8, –1.6)**	0.22
**Time in MVPA bouts ≥10 min**	–5.1 (–12.7, 3.3)	–4.8 (–8.3, –1.1)*	0.95

GDF-15, growth differentiation factor 15; LPA, light physical activity; MVPA, moderate-to-vigorous physical activity; PA, physical activity.

Values are mean percentage differences ([exponentiated differences in log-transformed values of GDF-15 – 1] x 100) per 30 min/day increment (95% confidence interval).

Linear regression model adjusted for sex, age, educational level (primary or less, secondary, or university), smoking status (never, former, or current), alcohol consumption (never, moderate, heavy, or former), energy intake (kcal/day), Mediterranean Diet Adherence Screener (MEDAS) score, body mass index (kg/m^2), serum glucose (mg/dL), serum LDL-cholesterol (mg/dL), systolic blood pressure (mmHg), cardiovascular disease (including acute myocardial infarction, stroke and congestive heart failure) and diabetes.

* *P* < 0.05.

** *P* < 0.01

*** *P* < 0.001.

[a] Including acute myocardial infarction, stroke and congestive heart failure.

[b] Low PA: total PA time ≤3.44 h/day; high PA: total PA time >3.44 h/day.

Table S3. Association of isotemporal replacement of activities with GDF-15 excluding participants with cardiovascular disease[a] or diabetes, stratified by total PA time

	**Participants with low PA time[b]** n=822	**Participants with high PA time[b]** n=932	**P for interaction**
**Sleep → SB**			
Model 1	0.6 (–0.5, 1.8)	0.2 (–1.1, 1.6)	0.68
Model 2	0.6 (–0.6, 1.7)	0.1 (–1.3, 1.4)	0.57
Model 3	0.5 (–0.7, 1.7)	0.1 (–1.3, 1.4)	0.62
**Sleep → LPA**			
Model 1	–4.2 (–7.0, –1.3)**	0.6 (–1.7, 3.0)	**0.009**
Model 2	–3.9 (–6.6, –1.0)**	0.3 (–2.0, 2.6)	**0.02**
Model 3	–3.9 (–6.6, –1.0)**	0.2 (–2.0, 2.5)	**0.02**
**Sleep → MVPA**			
Model 1	–8.0 (–12.2, –3.7)***	–2.8 (–5.3, –0.2)*	**0.03**
Model 2	–6.5 (–10.7, –2.1)**	–2.5 (–4.9, 0.0)	0.10
Model 3	–6.4 (–10.7, –2.0)**	–2.5 (–5.0, 0.0)	0.11
**SB → LPA**			
Model 1	–4.8 (–7.5, –2.0)**	0.4 (–1.7, 2.5)	**0.003**
Model 2	–4.4 (–7.2, –1.6)**	0.2 (–1.8, 2.3)	**0.007**
Model 3	–4.3 (–7.1, –1.5)**	0.2 (–1.9, 2.3)	**0.009**
**SB → MVPA**			
Model 1	–8.6 (–12.6, –4.4)***	–3.0 (–5.2, –0.8)**	0.02
Model 2	–7.1 (–11.1, –2.8)**	–2.5 (–4.7, –0.4)*	0.05
Model 3	–6.9 (–11.0, –2.7)**	–2.6 (–4.7, –0.4)*	0.06
**LPA → MVPA**			
Model 1	–4.0 (–9.6, 1.9)	–3.4 (–6.6, –0.1)*	0.85
Model 2	–2.8 (–8.4, 3.2)	–2.8 (–5.9, 0.5)	1.00
Model 3	–2.7 (–8.3, 3.3)	–2.7 (–5.9, 0.5)	0.99

GDF-15, growth differentiation factor 15; LPA, light physical activity; MVPA, moderate-to-vigorous physical activity; PA, physical activity; SB, sedentary behavior.

Values are mean percentage differences ([exponentiated differences in log-transformed values of GDF-15 – 1] x 100) per 30 min/day replacement (95% confidence interval).

Model 1: Linear regression model including total time (24 h) and all activities (sleep, SB, LPA, and MVPA) except the one being replaced, and adjusted for sex, age, and educational level (primary or less, secondary, or university).

Model 2: As model 1 and further adjusted for smoking status (never, former, or current), alcohol consumption (never, moderate, heavy, or former), energy intake (kcal/day) and Mediterranean Diet Adherence Screener (MEDAS) score.

Model 3: As model 2 and further adjusted for body mass index (kg/m^2), serum glucose (mg/dL), serum LDL-cholesterol (mg/dL), and systolic blood pressure (mmHg).

* $P < 0.05$.

** $P < 0.01$

*** $P < 0.001$.

[a] Including acute myocardial infarction, stroke and congestive heart failure.

[b] Low PA: total PA time ≤3.44 h/day; high PA: total PA time >3.44 h/day.

Table S4. Association of time spent in each activity with GDF-15, stratified by compliance with PA recommendations (≥30 min/day of MVPA)

	**Participants not meeting PA recommendations** n=474	**Participants meeting PA recommendations** n=1772	**P for interaction**
**Sleep**			
Model 1	0.7 (–0.9, 2.2)	–0.3 (–1.4, 0.8)	0.31
Model 2	0.5 (–1.1, 2.0)	–0.2 (–1.3, 0.8)	0.47
Model 3	0.8 (–0.6, 2.3)	–0.1 (–1.1, 0.9)	0.27
**SB**			
Model 1	1.9 (0.3, 3.5)***	1.5 (0.7, 2.4)***	0.71
Model 2	1.8 (0.3, 3.4)*	1.3 (0.5, 2.2)**	0.57
Model 3	0.9 (–0.6, 2.3)	0.8 (–0.0, 1.6)	0.92
**LPA**			
Model 1	–7.8 (–10.7, –4.9)***	–1.9 (–3.4, –0.5)**	**<0.001**
Model 2	–7.2 (–10.0, –4.2)***	–1.7 (–3.2, –0.3)*	**0.001**
Model 3	–5.2 (–7.9, –2.4)***	–0.9 (–2.2, 0.5)	**0.005**
**MVPA**			
Model 1	–39.9 (–48.5, –29.9)***	–4.4 (–6.2, –2.6)***	**<0.001**
Model 2	–37.4 (–46.3, –27.1)***	–3.9 (–5.7, –2.0)***	**<0.001**
Model 3	–29.8 (–39.1, –19.1)***	–2.7 (–4.4, –0.9)**	**<0.001**

GDF-15, growth differentiation factor 15; LPA, light physical activity; MVPA, moderate-to-vigorous physical activity; PA, physical activity; SB, sedentary behavior.

Values are mean percentage differences ([exponentiated differences in log-transformed values of GDF-15 – 1] x 100) per 30 min/day increment (95% confidence interval).

Model 1: Linear regression model adjusted for sex, age, and educational level (primary or less, secondary, or university).

Model 2: As model 1 and further adjusted for smoking status (never, former, or current), alcohol consumption (never, moderate, heavy, or former), energy intake (kcal/day) and Mediterranean Diet Adherence Screener (MEDAS) score.

Model 3: As model 2 and further adjusted for body mass index (kg/m^2), serum glucose (mg/dL), serum LDL-cholesterol (mg/dL), systolic blood pressure (mmHg), cardiovascular disease (including acute myocardial infarction, stroke and congestive heart failure) and diabetes.

* $P < 0.05$.

** $P < 0.01$

*** $P < 0.001$.

Table S5. Association of time accumulated in bouts of each activity with GDF-15, stratified by compliance with PA recommendations (≥30 min/day of MVPA)

	**Participants not meeting PA recommendations** n=474	**Participants meeting PA recommendations** n=1772	**P for interaction**
**Time in sedentary bouts ≥10 min**	1.4 (0.3, 2.4)*	0.4 (–0.1, 0.9)	0.11
**Time in sedentary bouts ≥30 min**	1.4 (0.5, 2.3)**	0.4 (–0.0, 0.9)	0.06
**Time in LPA bouts ≥1 min**	–8.8 (–15.0, –2.0)*	–2.0 (–5.0, 1.1)	0.07
**Time in LPA bouts ≥10 min**	–15.8 (–29.8, 0.9)	–8.7 (–16.6, 0.0)	0.43
**Time in MVPA bouts ≥1 min**	–43.7 (–56.6, –27.0)***	–4.2 (–6.4, –1.9)***	**<0.001**
**Time in MVPA bouts ≥10 min**	–31.7 (–66.1, 37.7)	–4.3 (–7.5, –0.9)*	0.35

GDF-15, growth differentiation factor 15; LPA, light physical activity; MVPA, moderate-to-vigorous physical activity; PA, physical activity.

Values are mean percentage differences ([exponentiated differences in log-transformed values of GDF-15 – 1] x 100) per 30 min/day increment (95% confidence interval).

Linear regression model adjusted for sex, age, educational level (primary or less, secondary, or university), smoking status (never, former, or current), alcohol consumption (never, moderate, heavy, or former), energy intake (kcal/day), Mediterranean Diet Adherence Screener (MEDAS) score, body mass index (kg/m^2), serum glucose (mg/dL), serum LDL-cholesterol (mg/dL), systolic blood pressure (mmHg), cardiovascular disease (including acute myocardial infarction, stroke and congestive heart failure) and diabetes.

* $P < 0.05$.

** $P < 0.01$

*** $P < 0.001$.

Table S6. Association of isotemporal replacement of activities with GDF-15, stratified by compliance with PA recommendations (≥30 min/day of MVPA)

	**Participants not meeting PA recommendations** n=474	**Participants meeting PA recommendations** n=1772	**P for interaction**
**Sleep → SB**			
Model 1	0.3 (–1.3, 2.0)	0.8 (–0.3, 1.9)	0.67
Model 2	0.5 (–1.1, 2.1)	0.6 (–0.4, 1.8)	0.85
Model 3	–0.2 (–1.6, 1.3)	0.4 (–0.6, 1.4)	0.51
**Sleep → LPA**			
Model 1	–2.5 (–6.4, 1.6)	0.1 (–1.7, 2.0)	0.24
Model 2	–2.2 (–6.1, 1.9)	0.1 (–1.7, 1.9)	0.28
Model 3	–1.5 (–5.2, 2.2)	0.5 (–1.2, 2.1)	0.33
**Sleep → MVPA**			
Model 1	–34.6 (–46.5, –20.1)***	–3.5 (–5.8, –1.1)**	**<0.001**
Model 2	–32.4 (–44.6, –17.4)***	–3.1 (–5.4, –0.7)*	**<0.001**
Model 3	–26.9 (–39.2, –12.2)**	–2.4 (–4.6, –0.2)*	**0.002**
**SB → LPA**			
Model 1	–2.8 (–6.9, 1.4)	–0.6 (–2.3, 1.1)	0.32
Model 2	–2.7 (–6.7, 1.5)	–0.6 (–2.2, 1.1)	0.35
Model 3	–1.4 (–5.1, 2.6)	0.0 (–1.5, 1.6)	0.50
**SB → MVPA**			
Model 1	–34.9 (–46.6, –20.5)***	–4.2 (–6.3, –2.1)***	**<0.001**
Model 2	–32.7 (–44.8, –18.0)***	–3.7 (–5.8, –1.6)**	**<0.001**
Model 3	–26.8 (–39.0, –12.2)**	–2.8 (–4.8, –0.8)**	**0.002**
**LPA → MVPA**			
Model 1	–33.0 (–46.6, –15.8)**	3.6 (–6.8. –0.3)*	**0.002**
Model 2	–30.9 (–44.9, –13.3)**	–3.1 (–6.3. 0.1)	**0.004**
Model 3	–25.8 (–39.8, 8.6)**	–2.9 (–5.8. 0.2)	**0.01**

GDF-15, growth differentiation factor 15; LPA, light physical activity; MVPA, moderate-to-vigorous physical activity; PA, physical activity; SB, sedentary behavior.

Values are mean percentage differences ([exponentiated differences in log-transformed values of GDF-15 – 1] x 100) per 30 min/day replacement (95% confidence interval).

Model 1: Linear regression model including total time (24 h) and all activities (sleep, SB, LPA, and MVPA) except the one being replaced, and adjusted for sex, age, and educational level (primary or less, secondary, or university).

Model 2: As model 1 and further adjusted for smoking status (never, former, or current), alcohol consumption (never, moderate, heavy, or former), energy intake (kcal/day) and Mediterranean Diet Adherence Screener (MEDAS) score.

Model 3: As model 2 and further adjusted for body mass index (kg/m^2), serum glucose (mg/dL), serum LDL-cholesterol (mg/dL), systolic blood pressure (mmHg), cardiovascular disease (including acute myocardial infarction, stroke and congestive heart failure) and diabetes.

* $P < 0.05$.

** $P < 0.01$

*** $P < 0.001$.
